# A hot water extract of turmeric (*Curcuma longa*) suppresses acute ethanol-induced liver injury in mice by inhibiting hepatic oxidative stress and inflammatory cytokine production

**DOI:** 10.1017/jns.2016.43

**Published:** 2017-01-12

**Authors:** Ryusei Uchio, Yohei Higashi, Yusuke Kohama, Kengo Kawasaki, Takashi Hirao, Koutarou Muroyama, Shinji Murosaki

**Affiliations:** 1 Research & Development Institute, House Wellness Foods Corporation, 3–20 Imoji, Itami 664-0011, Japan; 2 Central Research & Development Institute, House Foods Group Inc., 1–4 Takanodai, Yotsukaido 284-0033, Japan

**Keywords:** Turmeric (*Curcuma longa*), Bisacurone, Ethanol-induced liver injury, Oxidative stress, Inflammatory cytokines, ALT, alanine aminotransferase, AST, aspartate aminotransferase, BW, body weight, GSH, glutathione, GSSG, oxidised glutathione, O_2_^•−^, superoxide anion radical, ROS, reactive oxygen species, SOD, superoxide dismutase, TBARS, thiobarbituric acid-reactive substances, WEC, hot water extract of *Curcuma longa*

## Abstract

Turmeric (*Curcuma longa*) is a widely used spice that has various biological effects, and aqueous extracts of turmeric exhibit potent antioxidant activity and anti-inflammatory activity. Bisacurone, a component of turmeric extract, is known to have similar effects. Oxidative stress and inflammatory cytokines play an important role in ethanol-induced liver injury. This study was performed to evaluate the influence of a hot water extract of *C. longa* (WEC) or bisacurone on acute ethanol-induced liver injury. C57BL/6 mice were orally administered WEC (20 mg/kg body weight; BW) or bisacurone (60 µg/kg BW) at 30 min before a single dose of ethanol was given by oral administration (3·0 g/kg BW). Plasma levels of aspartate aminotransferase and alanine aminotransferase were markedly increased in ethanol-treated mice, while the increase of these enzymes was significantly suppressed by prior administration of WEC. The increase of alanine aminotransferase was also significantly suppressed by pretreatment with bisacurone. Compared with control mice, animals given WEC had higher hepatic tissue levels of superoxide dismutase and glutathione, as well as lower hepatic tissue levels of thiobarbituric acid-reactive substances, TNF-α protein and IL-6 mRNA. These results suggest that oral administration of WEC may have a protective effect against ethanol-induced liver injury by suppressing hepatic oxidation and inflammation, at least partly through the effects of bisacurone.

Alcohol is a popular beverage in most parts of the world and it has long been identified as a major risk factor for liver disease^(^^1^^)^, with excessive alcohol consumption causing impairment of both physical and mental health. The liver is the main site of ethanol metabolism and is also the principal target organ for ethanol-induced damage. Excessive ethanol consumption can trigger the progression of alcoholic liver disease, which covers a wide spectrum from steatosis to steatohepatitis, fibrosis and/or cirrhosis in severe cases^(^^2^^,^^3^^)^.

Oxidative stress is well known to play a key role in the pathogenesis of acute ethanol-induced liver injury^(^^3^^,^^4^^)^. Ethanol consumption induces excessive production of reactive oxygen species (ROS), which decrease hepatic tissue levels of superoxide dismutase (SOD) and glutathione (GSH), leading to overload of the antioxidant system and failure to efficiently remove ROS. As a result, hepatocyte necrosis and/or apoptosis are induced by oxidation of lipids, proteins and DNA^(^^5^^–^^7^^)^. Therefore, maintenance of hepatic antioxidant capacity is expected to alleviate ethanol-induced liver injury, and antioxidant therapy has been reported to prevent ethanol-induced liver damage^(^^7^^–^^9^^)^.

Clinical and animal studies have revealed that inflammatory cytokines such as TNF-α and IL-6 are key mediators of ethanol-induced liver injury^(^^10^^,^^11^^)^. TNF-α was reported to induce hepatocyte apoptosis and liver injury *in vivo* via a cathepsin B-mediated pathway^(^^12^^)^. It was also reported that reduction of TNF-α and IL-6 levels by suppression of oxidative activity can alleviate ethanol-induced liver inflammation^(^^13^^)^.

Turmeric (*Curcuma longa*) is a widely used spice that possesses various biological activities^(^^14^^,^^15^^)^. For example, aqueous extracts of turmeric have been reported to exhibit antioxidant activity^(^^16^^)^ and anti-inflammatory activity^(^^17^^)^, as well as promoting corneal wound healing^(^^18^^)^, an antidepressant effect^(^^19^^)^, an anticancer effect^(^^20^^)^ and regulating cytochrome P450 (CYP) activity^(^^21^^)^. We recently reported that a hot water extract of *C. longa* (WEC) modulates the adhesive properties of endothelial cells by suppressing TNF-α-induced expression of cell adhesion molecules via inhibition of the NF-κB signalling pathway^(^^22^^)^. These effects of WEC are at least partly attributable to bisacurone, a component of turmeric that has both antioxidant and anti-inflammatory activities^(^^17^^,^^23^^)^. However, the influence of WEC or bisacurone on ethanol-induced liver injury has not yet been investigated.

Accordingly, the present study was performed to determine the effects of oral administration of WEC or bisacurone on ethanol-induced liver injury in mice by examining plasma markers of liver damage. We also assessed the effects of WEC on hepatic oxidation and inflammation in ethanol-treated mice.

## Materials and methods

### Preparation of a hot water extract of *Curcuma longa*

WEC was prepared according to the method described previously^(^^22^^)^. In brief, rhizomes of turmeric (*Curcuma longa* Linn.) were extracted with hot water at 95°C, after which the supernatant fraction was concentrated under reduced pressure and WEC powder was obtained by spray drying. This powder was stored at 4°C until use. WEC powder had a bisacurone content of 0·302 % (w/w) and a curcumin content of 0·125 % (w/w).

### Preparation of bisacurone

WEC was incubated with methanol–water (90:10) and the extract was freeze-dried. Then the freeze-dried powder was dissolved in acetonitrile–water (30:70) and subjected to preparatory reverse-phase HPLC (YMC ODS-A-HG column (YMC Co.), mobile phase: acetonitrile–water (35:65)). The fraction containing bisacurone was concentrated and dissolved in ethyl acetate–hexane (80:20), after which the resulting solution was subjected to silica gel open column chromatography (YMC GEL SIL-HG; YMC Co.). Next, the eluate was concentrated and dissolved in ethyl acetate–chloroform (64:36), following which the resulting solution was applied to a preparatory normal-phase MPLC system (ULTRA PACK SI-40B column, mobile phase: ethyl acetate–chloroform (64:36 to 38:62)). After the fraction containing bisacurone was concentrated, it was dissolved in acetonitrile–water (30:70) and the resulting solution was applied to a preparatory reverse-phase HPLC system (ULTRON VX-ODS column, mobile phase: acetonitrile–water (30:70)). Subsequently, the fraction containing bisacurone was concentrated and extracted with chloroform–water (35:65), after which the chloroform layer was dried and concentrated to obtain bisacurone. The bisacurone content of this final material was 83·6 %, as determined by quantitative NMR^(^^24^^)^.

### Animals

Specific-pathogen-free male C57BL/6N CrlCrlj mice were purchased from Charles River Japan and were acclimatised for 7 d on the basal diet before experiments were performed. The basal diet was based on the American Institute of Nutrition (AIN)-93G diet^(^^25^^)^. α-Maize starch, casein, soyabean oil, cellulose powder, AIN-93G mineral mixture, and AIN-93 vitamin mixture were purchased from Oriental Yeast Co. Maize starch and sucrose were obtained from Matsutani Chemical Industry and Mitsui Sugar Co., Ltd., respectively. Choline bitartrate, l-cystine and *tert*-butylhydroquinone (TBHQ) were purchased from Wako Pure Chemicals. Throughout the experiments, mice were housed individually in cages and maintained under specific-pathogen-free conditions in a controlled environment (room temperature: 23 ± 1°C, relative humidity: 55 ± 5 %, and 12 h light–12 h dark cycle). All experiments were performed with 9-week-old male C57BL/6N mice (19–22 g) in accordance with the guidelines of the Animal Care and Use Committee of the House Wellness Foods Corporation.

### Experimental design

C57BL/6N mice were allocated to a control group and a WEC (20 mg/kg body weight (BW)) group or to a control group and a bisacurone (60 µg/kg BW) group so that the BW of each group was balanced. The group size for these experiments was determined as follows. Our preliminary study revealed that the mean plasma alanine aminotransferase (ALT) level was approximately 11 (sd 2) IU/l at 6 h after administration of a single dose of ethanol (3·0 g/kg BW) to C57BL/6N mice. In addition, the antioxidant *N*-acetylcysteine was reported to inhibit elevation of the plasma ALT level (by about 40 % *v.* the control group) at 6 h after administration of ethanol to mice^(^^9^^)^. Based on an expected mean plasma ALT level of 11 (sd 2) IU/l at 6 h after ethanol administration and a targeted 40 % reduction of plasma ALT by WEC, a group size of six mice was estimated to give the study a statistical power of 80 % with a type I error of 5 %. Mice were orally administered WEC at the dose of 20 mg/kg BW in the WEC group and received bisacurone at the dose of 60 µg/kg BW in the bisacurone group, while the respective control groups were given the same dose of the vehicle (0·5 % (w/v) methylcellulose in water (Wako Pure Chemical Industries)). Ethanol was orally administered to the mice (3·0 g/kg BW and 200 µl/20 g BW) as a 15 % (w/v) solution in water at 30 min after administration of WEC, bisacurone or the vehicle. Plasma aspartate aminotransferase (AST) and ALT levels were measured immediately before and 1, 2, 4 and 6 h after ethanol administration in all experiments. Hepatic tissue levels of SOD, GSH, the GSH:oxidised-GSH (GSSG) ratio, thiobarbituric acid-reactive substances (TBARS), TNF-α protein and mRNA, and IL-6 mRNA were also measured at 1, 2, 4 and 6 h after ethanol administration. These parameters were measured in untreated control mice that did not receive WEC or ethanol (*n* 12), as well as in the ethanol-treated control group and the ethanol-treated WEC group (both *n* 6). Blood samples were collected from the retro-orbital sinus into heparinised calibrated pipettes (Drummond Scientific Company). Mice were anaesthetised with diethyl ether immediately before being killed by exsanguination, after which their livers were harvested and washed with saline to minimise contamination by blood.

### Measurement of plasma aspartate aminotransferase and alanine aminotransferase

Blood samples were centrifuged (12 000 ***g*** for 10 min at 4°C) immediately after collection to obtain plasma. Then AST and ALT were measured by the pyruvate oxidase-*N*-ethyl-*N*-(2-hydroxy-3-sulfopro-pyl)-*m*-toluidine (POP-TOOS) method with commercial kits (Transaminase CII-test Wako; Wako Pure Chemical) according to the manufacturer's instructions^(^^26^^,^^27^^)^.

### Hepatic histological analysis

Liver tissue specimens were fixed in 10 % (v/v) neutral buffered formalin (Wako Pure Chemical), dehydrated in an ethanol series, cleared in xylene and embedded in paraffin. The paraffin blocks were cut into sections approximately 5 µm thick, which were defatted with xylene and stained with haematoxylin and eosin (H&E) (Merck)^(^^28^^,^^29^^)^. Sections were viewed under an inverted microscope (Olympus IX-73; Olympus) (original magnification × 160).

### Measurement of hepatic superoxide dismutase activity

Liver tissue (30 mg) was homogenised in eight volumes of sucrose buffer (0·25 m-sucrose, 10 mm-Tris(hydroxymethyl)aminomethane (Tris), 1 mm-EDTA, pH 7·40) using a disposable homogeniser (BioMasher II; Nippi Inc.). The homogenate was sonicated once with a Sonifire SLPe 40 (Branson) for 3 s at 20 % amplitude on ice and then centrifuged (10 000 ***g*** for 60 min at 4°C), after which the supernatant was stored at  −80°C until use. SOD activity was measured by the water-soluble tetrazolium salt (WST) method using a SOD assay kit-WST (Dojindo Inc.), according to the manufacturer's instructions^(^^30^^,^^31^^)^. One unit (U) of SOD activity was defined as causing 50 % inhibition of the assay reaction and hepatic SOD activity was normalised per g liver tissue (wet weight).

### Measurement of the hepatic glutathione level and glutathione:oxidised glutathione ratio

Liver tissue (100 mg) was added to 10 volumes of 5 % (w/v) 5-sulfosalicyclic acid (SSA) solution and was homogenised with a disposable homogeniser. The homogenate was centrifuged at 8000 ***g*** for 10 min at 4°C, after which the supernatant was diluted 10-fold with deionised water and stored at −80°C until use. Total GSH and GSSG levels were determined by the enzymic cycling method with 5,5-dithio-bis(2-nitrobenzoic acid) (DTNB) using a GSSG/GSH Quantification Kit (Dojindo Molecular Technologies Inc.), according to the manufacturer's instructions^(^^32^^,^^33^^)^. Then the GSH level was calculated from the difference between total GSH and GSSG, and the GSH:GSSG ratio was also calculated. Both the hepatic GSH level and GSH:GSSG ratio were normalised per g liver tissue (wet weight).

### Measurement of hepatic lipid peroxides

The hepatic tissue level of TBARS was measured as a marker of lipid peroxidation. Liver tissue (20 mg) was added to 10 volumes of radioimmunoprecipitation (RIPA) buffer (250 mm-Tris-HCl, pH 7·6, 750 mm-sodium chloride, 5 % Tergitol (NP-40), 2·5 % sodium deoxycholate, 0·5 % SDS; Cayman Chemical) supplemented with protease inhibitor cocktail (Sigma-Aldrich) and was homogenised with a disposable homogeniser. The homogenate was sonicated twice with a Sonifire SLPe 40 for 3 s at 20 % amplitude on ice and centrifuged (1600 ***g*** for 10 min at 4°C), after which the supernatant fraction was stored at −80°C until use. TBARS were determined by fluorometric measurement of malondialdehyde and thiobarbituric acid (MDA-TBA) adducts using a TBARS assay kit (Cayman Chemical) according to the manufacturer's protocol^(^^34^^,^^35^^)^, and the hepatic TBARS level was normalised per g liver tissue (wet weight).

### Measurement of hepatic TNF-α protein

Liver tissue (200 mg) was added to 2·5 volumes of lysis buffer (CelLytic™ MT; Sigma-Aldrich) supplemented with a protease inhibitor cocktail (Sigma-Aldrich) and was homogenised by using a disposable homogeniser. The homogenate was sonicated once with a Sonifire SLPe 40 for 3 s at 20 % amplitude on ice and centrifuged (16 000 ***g*** for 10 min at 4°C), after which the supernatant fraction was stored at −80°C until use. TNF-α protein was determined by a sandwich ELISA using the Quantikine^®^ mouse TNF-α ELISA kit (R&D Systems) according to the manufacturer's instructions^(^^36^^–^^38^^)^, and the hepatic TNF-α level was normalised per g liver tissue (wet weight).

### Measurement of hepatic TNF-α and IL-6 mRNA expression

After RNAlater^®^ (Ambion Inc.) solution (300 µl) was added to liver tissue (30 mg) to prevent degradation of mRNA, the tissue samples were stored at −80°C until use. Total RNA was prepared by using the RNeasy^®^ Mini Kit (Qiagen), and DNA was removed by on-column DNase digestion with an RNase-free DNase Set (Qiagen) according to the manufacturer's protocol. Then expression of TNF-α, IL-6 and β-actin mRNA was measured by real-time PCR^(^^39^^)^. In brief, synthesis of cDNA and PCR were performed using the Thermal Cycler Dice^®^ Real Time System TP800 (Takara) and One Step SYBR^®^ PrimeScript™ RT-PCR Kit II (Takara) according to the manufacturer's instructions. The specific primer for TNF-α was obtained from Life Technologies, Inc., while the primers for IL-6 and β-actin were obtained from Takara. Primer sequences were as follows: TNF-α (forward primer 5′-CCTGTAGCCCACGTCGTAG-3′, reverse primer; 5′-GGGAGTAGACAAGGTACAACCC-3′), IL-6 (forward primer 5′-CCACTTCACAAGTCGGGAGGCTTA-3′, reverse primer; 5′-CCAGTTTGGTAGCATCCATCATTTC-3′) and β-actin (forward primer 5′-GGCTGTATTCCCCTCCATCG-3′, reverse primer; 5′-CCAGTTGGTAACAATGCCATGT-3′). Data were analysed by the 2^−ΔΔCT^ method^(^^40^^)^ using the second derivative curve of amplification plots (Thermal Cycler Dice Real Time System software version 4.00B; Takara). Expression of TNF-α and IL-6 mRNA was normalised for β-actin mRNA expression.

### Statistical analysis

Differences between two groups were assessed with Student's unpaired *t* test. Data were also analysed by one-way ANOVA, followed by the Tukey–Kramer test, for comparison between the untreated control group and the ethanol-treated control group. All analyses were performed using Statcel 3 software (OMS Publishing). Results are shown as mean values and standard deviations. *P* < 0·05 was considered to indicate statistical significance.

## Results

### Effect of hot water extract of *Curcuma longa* on plasma aspartate aminotransferase and alanine aminotransferase levels after acute ethanol administration

**Fig. 1.**
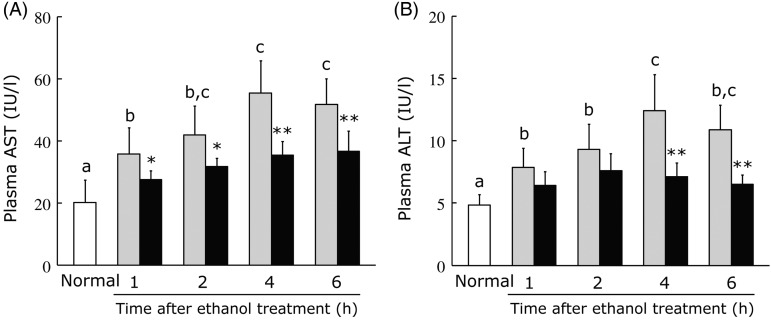
Effects of oral administration of hot water extract of *Curcuma longa* (WEC) on plasma liver enzymes after a single dose of ethanol (3·0 g/kg body weight) in mice. Mice were given vehicle (░) or WEC (■) prior to ethanol administration. Plasma aspartate aminotransferase (AST) (A) and alanine aminotransferase (ALT) (B) levels were measured immediately before (□) and after the ethanol administration. Values are means for *n* 6 (control and WEC groups) or *n* 12 (normal group), with standard deviations represented by vertical bars. ^a,b,c^ For bars accompanied by letters, mean values with unlike letters were significantly different (*P* < 0·05; one-way ANOVA, *post hoc* Tukey–Kramer test). Mean value was significantly different from that of the control group: * *P* < 0·05, ** *P* < 0·01 (unpaired Student's *t* test). IU, international units.

Because aqueous extracts of turmeric have been reported to protect the liver from injury by carbon tetrachloride^(^^41^^)^, we evaluated the effect of WEC on ethanol-induced liver injury. Mice were orally administered the vehicle or WEC (20 mg/kg), and a single dose of ethanol (3·0 g/kg) was given after 30 min. In the control group, plasma AST and ALT levels were markedly increased at 1, 2, 4 and 6 h after ethanol administration. In the WEC group, the plasma AST level was significantly lower at 1, 2, 4 and 6 h after ethanol administration compared with that in the control group (Fig. 1(A)). Plasma ALT was also significantly lower at 4 and 6 h in the WEC group compared with the control group (Fig. 1(B)).

### Effect of bisacurone on plasma aspartate aminotransferase and alanine aminotransferase levels after acute ethanol administration

**Fig. 2.**
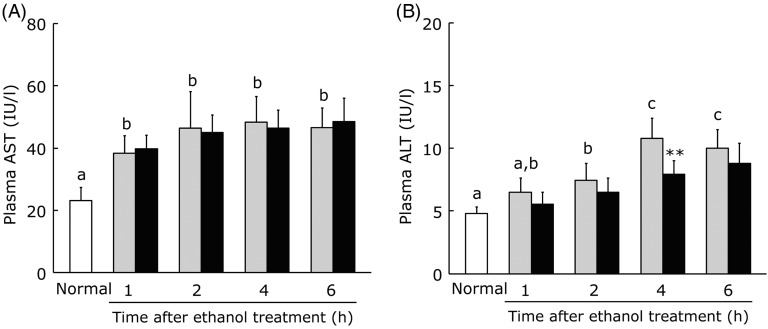
Effects of oral administration of bisacurone on plasma liver enzymes after a single dose of ethanol (3·0 g/kg body weight) in mice. Mice were given vehicle (░) or bisacurone (■) prior to ethanol administration. Plasma aspartate aminotransferase (AST) (A) and alanine aminotransferase (ALT) (B) levels were measured immediately before (□) and after the ethanol administration. Values are means for for *n* 6 (control and bisacurone groups) or *n* 12 (normal group), with standard deviations represented by vertical bars. ^a,b,c^ For bars accompanied by letters, mean values with unlike letters were significantly different (*P* < 0·05; one-way ANOVA, *post-hoc* Tukey–Kramer test). ** Mean value was significantly different from that of the control group (*P* < 0·01; unpaired Student's *t* test). IU, international units.

Bisacurone is a component of turmeric extract with both antioxidant and anti-inflammatory activities^(^^17^^,^^23^^)^. Therefore, we also evaluated the effect of pretreatment with bisacurone on ethanol-induced liver injury when it was given to mice at a dose corresponding to the bisacurone content in WEC. Mice were orally administered the vehicle or bisacurone (60 µg/kg), and a single dose of ethanol (3·0 g/kg) was given after 30 min. In the control group, plasma AST and ALT levels showed a marked increase at 1, 2, 4 and 6 h after ethanol administration. While the plasma AST level showed no significant difference between the control group and the bisacurone group (Fig. 2(A)), plasma ALT was significantly lower at 4 h after ethanol administration in the bisacurone group compared with the control group (Fig. 2(B)).

### Effect of hot water extract of *Curcuma longa* on hepatic histological changes after acute ethanol administration

**Fig. 3.**
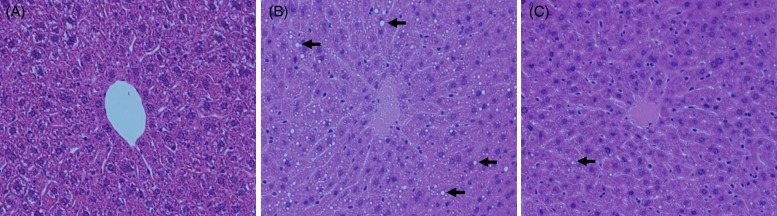
Effect of hot water extract of *Curcuma longa* (WEC) on hepatic histological changes after a single dose of ethanol (3·0 g/kg body weight) in mice. Mice were administered WEC or the vehicle prior to ethanol. Liver histology was examined before and 6 h after ethanol administration. (A) Normal; (B) control group; (C) WEC group. →, Lipid droplets. Haematoxylin and eosin stain; original magnification × 160.

Ingestion of ethanol causes acute histological changes of the liver such as microvesicular steatosis^(^^9^^,^^37^^)^. Accordingly, we examined hepatic histology in mice before and 6 h after administration of ethanol (3·0 g/kg) with or without WEC pretreatment. In contrast to normal mice (Fig. 3(A)), lipid droplets (microvesicular steatosis) were observed in the control group after ethanol administration (Fig. 3(B)). The changes were milder in the WEC group, with small lipid droplets being observed after ethanol administration (Fig. 3(C)).

### Effect of hot water extract of *Curcuma longa* on hepatic superoxide dismutase, glutathione, glutathione:oxidised glutathione ratio and thiobarbituric acid-reactive substances after acute ethanol administration

**Fig. 4.**
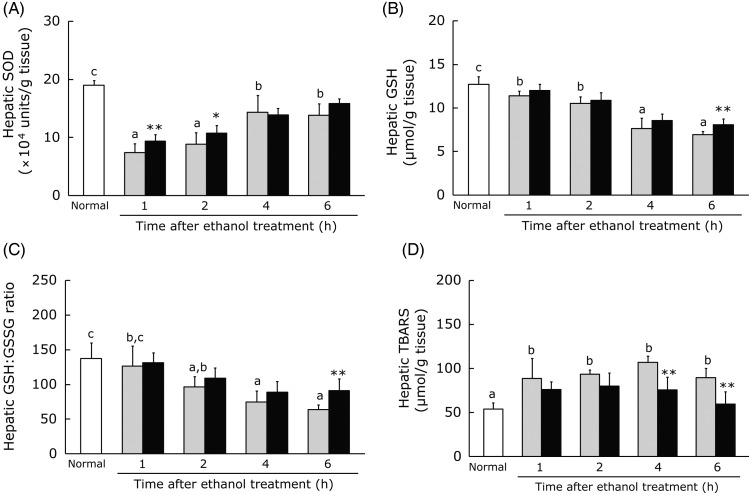
Effects of oral administration of hot water extract of *Curcuma longa* (WEC) on hepatic antioxidant activities and hepatic lipid peroxide content after a single dose of ethanol (3·0 g/kg body weight) in mice. Mice were given vehicle (░) or WEC (■) prior to ethanol administration. Hepatic superoxide dismutase (SOD) activity (A), glutathione (GSH) level (B), glutathione:oxidised glutathione (GSH:GSSG) ratio (C) and thiobarbituric acid-reactive substances (TBARS) (D) were measured immediately before (□) and after the ethanol administration. Values are means for *n* 6 (control and WEC groups) or *n* 12 (normal group), with standard deviations represented by vertical bars. ^a,b,c^ For bars accompanied by letters, mean values with unlike letters were significantly different (*P* < 0·05; one-way ANOVA, *post hoc* Tukey–Kramer test). Mean value was significantly different from that of the control group: * *P* < 0·05, ** *P* < 0·01 (unpaired Student's *t* test).

Acute ethanol intake leads to elevation of hepatic lipid peroxidation markers such as TBARS due to consumption of antioxidants such as GSH and depression of SOD activity^(^^42^^)^. We measured the hepatic tissue SOD activity, GSH level, GSH:GSSG ratio and TBARS level in mice administered ethanol (3·0 g/kg) at 30 min after receiving WEC or the vehicle. In the control group, hepatic SOD activity showed a significant decrease at 1, 2, 4 and 6 h after ethanol administration, while it was significantly higher at 1 and 2 h in the WEC group compared with the control group (Fig. 4(A)). In addition, the hepatic GSH level and GSH:GSSG ratio were both significantly decreased at 1, 2, 4 and 6 h after ethanol administration in the control group, while these parameters were significantly higher at 6 h in the WEC group compared with the control group (Fig. 4(B) and (C)). Furthermore, the hepatic TBARS level showed a significant increase at 1, 2, 4 and 6 h after ethanol administration in the control group, whereas it was significantly lower at 4 and 6 h in the WEC group compared with the control group (Fig. 4(D)).

### Effect of hot water extract of *Curcuma longa* on hepatic TNF-α protein production and expression of TNF-α and IL-6 mRNA after acute ethanol administration

**Fig. 5.**
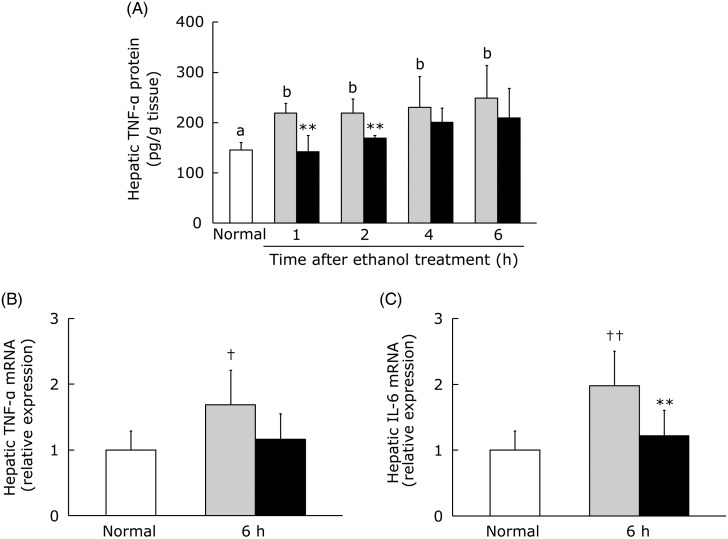
Effect of oral administration of hot water extract of *Curcuma longa* (WEC) on hepatic inflammatory cytokines after a single dose of ethanol (3·0 g/kg body weight) in mice. Mice were given vehicle (░) or WEC (■) prior to ethanol administration. Hepatic TNF-α protein content (A), TNF-α mRNA level (B) and IL-6 mRNA level (C) were measured immediately before (□) and after the ethanol administration. The mRNA levels were quantified by using β-actin as the internal standard. Protein values are means for *n* 6 (control and WEC groups) or *n* 12 (normal group), with standard deviations represented by vertical bars. mRNA values are means for *n* 6 (normal, control and WEC groups), with standard deviations represented by vertical bars. ^a,b^ For bars accompanied by letters, mean values with unlike letters were significantly different (*P* < 0·05; one-way ANOVA, *post hoc* Tukey–Kramer test). ** Mean value was significantly different from that of the control group (*P* < 0·01; unpaired Student's *t* test). Mean value was significantly different from that of the normal group: † *P* < 0·05, †† *P* < 0·01 (unpaired Student's *t* test).

Acute ethanol administration was reported to increase hepatic levels of TNF-α protein, IL-6 protein and IL-6 mRNA^(^^37^^,^^43^^)^. Accordingly, we measured the hepatic TNF-α protein level and TNF-α and IL-6 mRNA expression in mice given ethanol (3·0 g/kg) at 30 min after administration of WEC or the vehicle. A significant increase of the hepatic TNF-α protein level was found in the control group at 1, 2, 4 and 6 h after ethanol administration, while TNF-α protein was significantly lower at 1 and 2 h in the WEC group compared with the control group (Fig. 5(A)). Hepatic TNF-α mRNA and IL-6 mRNA expression did not increase until 4 h after ethanol administration in the control group (data not shown), but a significant increase was detected at 6 h. TNF-α mRNA expression was lower in the WEC group than the control group at 6 h after ethanol administration (*P* = 0·077), and hepatic IL-6 mRNA expression was significantly lower in the WEC group at 6 h (Fig. 5(B) and (C)).

## Discussion

In the present study, WEC significantly prevented acute ethanol-induced liver injury, which was detected by elevation of plasma AST and ALT levels. In addition to the increase of plasma AST and ALT, mice given ethanol (3·0 g/kg) displayed a decrease of hepatic SOD activity, hepatic GSH level, and hepatic GSH:GSSG ratio, as well as an increase in hepatic TBARS level, hepatic TNF-α protein production and hepatic IL-6 mRNA expression. These changes due to administration of ethanol were significantly suppressed by pretreatment with WEC. In addition, we demonstrated that pretreatment with bisacurone significantly suppressed the elevation of plasma ALT after ethanol administration. Our findings suggest that WEC protects against ethanol-induced liver injury by maintaining hepatic antioxidant capacity, inhibiting hepatic lipid peroxidation, and inhibiting inflammatory cytokine production, with these effects being partly mediated through the actions of bisacurone.

The serum levels of AST and ALT reflect hepatocyte damage. AST is found in high concentrations in the liver, heart, skeletal muscle and kidneys, whereas ALT is more abundant in the liver than in other tissues. Therefore, ALT is thought to be more sensitive for detecting hepatocellular injury and is more specific to the liver than AST. However, it has been reported that AST increases preferentially in patients with alcoholic liver injury and there is only mild elevation of ALT^(^^44^^)^. In contrast, we found that both plasma AST and ALT were similarly elevated in the control group after mice were given a single dose of ethanol (Fig. 1(A) and (B)). Although it is uncertain which is a more reliable marker of ethanol-induced liver injury, elevation of both enzymes was significantly suppressed by WEC.

Ethanol-induced oxidative stress is known to play an important role in liver injury. Metabolism of ethanol via cytochrome P450 2E1 (CYP2E1) is an alternative pathway involving production of superoxide anion radicals (O_2_^•−^)^(^^7^^)^. SOD can convert O_2_^•−^ into H_2_O_2_, but its activity is inhibited by an excess of O_2_^•−^ and H_2_O_2_, suggesting that the level of SOD activity is an indicator of the severity of oxidative stress^(^^3^^,^^7^^,^^45^^)^. In fact, it has been reported that infusion of ethanol increases hepatic O_2_^•−^ production in rats^(^^46^^)^, while production of ROS by metabolism of ethanol leads to inactivation of SOD^(^^47^^)^. In accordance with these observations, we found that acute ethanol administration led to marked reduction of hepatic SOD activity in the control group. Aqueous extracts of turmeric have been reported to suppress *in vitro* O_2_^•−^ production at 2 h after exposure to pyrogallol, an O_2_^•−^ generator^(^^48^^)^, and also inhibit the decrease of myocardial SOD activity induced by ischaemia–reperfusion in rats^(^^49^^,^^50^^)^. Similar to these observations, we demonstrated that WEC inhibited the decrease of SOD activity after ethanol administration, probably by suppressing O_2_^•−^ production. Alleviation of oxidative stress by WEC was confirmed because it inhibited the decrease of both hepatic tissue GSH and the GSH:GSSG ratio in mice treated with ethanol (Fig. 4(B) and (C)) and also significantly reduced the elevation of hepatic TBARS induced by ethanol (Fig. 4(D)). These results suggest that WEC maintains sufficient hepatic antioxidant activity to inhibit an increase of lipid peroxidation and ameliorate liver injury after acute ethanol administration.

Both clinical and animal studies have revealed that inflammatory cytokines such as TNF-α and IL-6 are key mediators of ethanol-induced liver injury^(^^10^^,^^11^^)^, with apoptosis being induced by TNF-α and progression of hepatic inflammation being caused by both TNF-α and IL-6. We observed that WEC inhibited elevation of the hepatic TNF-α protein level in the early period after ethanol administration (Fig. 5(A)). Antioxidant treatment was reported to suppress induction of TNF-α protein in the liver at 1·5 h after acute ethanol administration in mice, possibly by suppressing ROS production^(^^37^^,^^51^^)^. In the present study, SOD activity remained high in the WEC group at 1 to 2 h after ethanol administration (Fig. 4(A)), suggesting that preservation of hepatic antioxidant activity by WEC may suppress induction of TNF-α protein production in the liver after ethanol administration. Activation of TNF-α signalling induces both TNF-α and IL-6 gene expression via the NF-κB signalling pathway^(^^52^^)^. Therefore, the increase of hepatic TNF-α and IL-6 mRNA expression at 6 h after ethanol administration that we detected in the present study was presumably related to elevation of hepatic TNF-α protein production. Accordingly, suppression of the increase in hepatic TNF-α protein after ethanol administration by pretreatment with WEC (Fig. 5(A)) could be associated with less induction of TNF-α and IL-6 mRNA in ethanol-treated mice (Fig. 5(B) and (C)). However, the possibility that expression of these mRNAs was reduced by inhibition of the NF-κB signalling pathway cannot be excluded.

Our previous study provided evidence that the anti-inflammatory activity of WEC, which inhibits the NF-κB signalling pathway, is partly due to a component called bisacurone^(^^22^^)^. Bisacurone has been found to suppress elevation of ROS, activation of NF-κB and expression of vascular cell adhesion molecules^(^^17^^)^. The present study showed that pretreatment with WEC provided protection against ethanol-induced liver injury along with maintenance of antioxidant activity and suppression of the up-regulation of TNF-α and IL-6 mRNA expression. Pretreatment with bisacurone also significantly suppressed the increase of ALT after ethanol administration (Fig. 2(B)), suggesting that the effects of WEC could be at least partly attributable to bisacurone and that it will be important to investigate the molecular mechanisms underlying the protective effect of bisacurone against ethanol-induced liver injury. In addition, since bisacurone pretreatment has a minor protective effect against ethanol-induced liver injury compared with the WEC effect, WEC components other than bisacurone may also be involved in the effect of WEC.

**Fig. 6.**
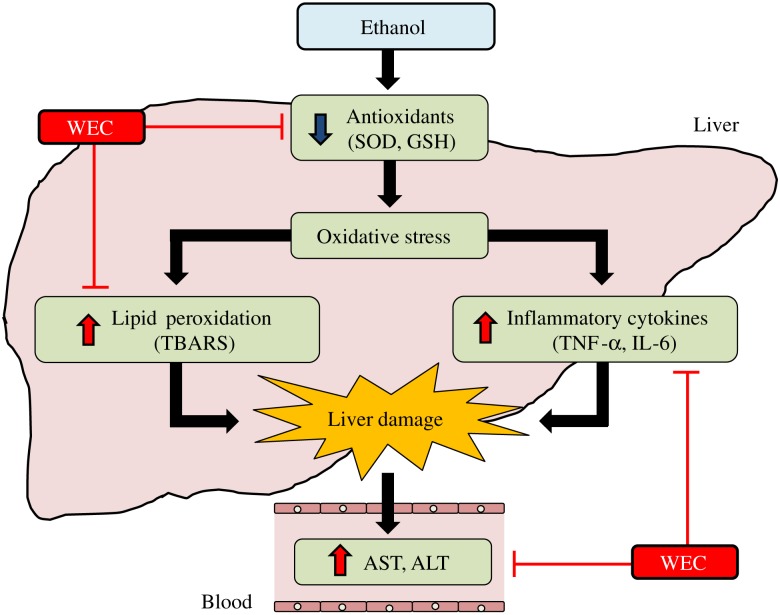
Graphical summary of the effect of hot water extract of *Curcuma longa* (WEC) on acute ethanol-induced liver injury in mice. Pretreatment with WEC maintained hepatic antioxidant activity, inhibited lipid peroxidation and inhibited inflammatory cytokine production after acute ethanol administration, resulting in the prevention of acute ethanol-induced liver injury in mice. SOD, superoxide dismutase; GSH, glutathione; TBARS, thiobarbituric acid-reactive substances; AST, aspartate aminotransferase; ALT, alanine aminotransferase.

In conclusion, we demonstrated that pretreatment with WEC maintained hepatic antioxidant activity, inhibited lipid peroxidation and inhibited inflammatory cytokine production after acute ethanol administration, resulting in the prevention of acute ethanol-induced liver injury in mice (Fig. 6). These findings suggest that a hot water extract of turmeric has the potential to provide effective protection against ethanol-induced liver damage.
